# Functional Analysis of Deep Intronic SNP rs13438494 in Intron 24 of *PCLO* Gene

**DOI:** 10.1371/journal.pone.0076960

**Published:** 2013-10-22

**Authors:** Seunghee Seo, Kanako Takayama, Kyosuke Uno, Kazutaka Ohi, Ryota Hashimoto, Daisuke Nishizawa, Kazutaka Ikeda, Norio Ozaki, Toshitaka Nabeshima, Yoshiaki Miyamoto, Atsumi Nitta

**Affiliations:** 1 Department of Pharmaceutical Therapy and Neuropharmacology, Faculty of Pharmaceutical Sciences, University of Toyama, Toyama, Japan; 2 Department of Pharmaceutical Education, Faculty of Pharmaceutical Sciences, University of Toyama, Toyama, Japan; 3 Department of Psychiatry, Osaka University Graduate School of Medicine, Osaka, Japan; 4 Molecular Research Center for Children’s Mental Development, United Graduate School of Child Development, Osaka University, Osaka, Japan; 5 Addictive Substance Project, Tokyo Metropolitan Institute of Medical Science, Tokyo, Japan; 6 Department of Psychiatry, Graduate School of Medicine, Nagoya University, Nagoya, Japan; 7 Department of Regional Pharmaceutical Care and Science, Meijo University, Nagoya, Japan; University of Illinois at Chicago, United States of America

## Abstract

The single nucleotide polymorphism (SNP) rs13438494 in intron 24 of *PCLO* was significantly associated with bipolar disorder in a meta-analysis of genome-wide association studies. In this study, we performed functional minigene analysis and bioinformatics prediction of splicing regulatory sequences to characterize the deep intronic SNP rs13438494. We constructed minigenes with A and C alleles containing exon 24, intron 24, and exon 25 of *PCLO* to assess the genetic effect of rs13438494 on splicing. We found that the C allele of rs13438494 reduces the splicing efficiency of the *PCLO* minigene. In addition, prediction analysis of enhancer/silencer motifs using the Human Splice Finder web tool indicated that rs13438494 induces the abrogation or creation of such binding sites. Our results indicate that rs13438494 alters splicing efficiency by creating or disrupting a splicing motif, which functions by binding of splicing regulatory proteins, and may ultimately result in bipolar disorder in affected people.

## Introduction

An important role of genetic factors in mental disorders was indicated by family linkage, twin, and adoption studies [1]–[4]. Genetic studies of mental disorders have been conducted to identify candidate genes, which hold the promise of improving our understanding of the neurobiological basis of mental disorders and may lead to the development of novel therapeutic and protective strategies [5].

In such an effort to search a gene that related to mental disorders, *PCLO* was identified as an overexpressed gene in the nucleus accumbens of mice subjected to repeated methamphetamine treatment, which can cause severe mental disorders [6]. *PCLO* regulates methamphetamine-induced behavioral sensitization and depression-like behavior [7], [8]. In addition, *PCLO* showed a selective increase in expression of NAc in behaviorally sensitized mice induced by repeated METH treatment, rather than a global increase in the brain [7]. Genome-wide association studies (GWASs) of major depressive disorder in humans also identified *PCLO* as a putative candidate gene [9]. The reanalysis of *PCLO* replication studies and meta-analyses provided evidence of an association of major depressive disorder with the single nucleotide polymorphism (SNP) rs2522833 in the *PCLO* region, indicating that *PCLO* may be a casual factor for major depression [10]–[12]. Moreover, a recent study identified 45 SNPs that were associated with the differential expression of genes in the prefrontal cortex of individuals with bipolar disorder [13]. One of the identified SNPs, rs13438494 in an intron of *PCLO*, was significantly associated with bipolar disorder in a large-scale meta-analysis of GWASs [13]. The difference in frequency for the risk allele in patients relative to control subjects is clearly too small to account for the differences in expression between the patients and the control subjects [13], [14]. In the allele frequency obtained within the NCBI SNP database (http://www.ncbi.nlm.nih.gov/snp) for studies representing diverse ethnic groups from Europe, Africa, Japan and China, the rs13438494 has the minor allele frequency of 0.322 (A allele).

In this study, we focused on rs13438494, a mutation in intron 24 of *PCLO*, which encodes a presynaptic cytomatrix protein and is important in monoaminergic neurotransmission in the brain [7]. The SNP rs13438494 in *PCLO* has not been characterized functionally. Therefore, in the present study, we conducted *in vitro* and *in silico* analysis of rs13438494 to confirm the effect of this allele on splicing. Our results demonstrate that rs13438494 alters the splicing efficiency by creating or disrupting a splicing motif that functions by binding of the splicing regulatory protein and may ultimately influence bipolar disorder.

## Materials and Methods

### Construction of *PCLO* Minigenes

**Figure 1:**
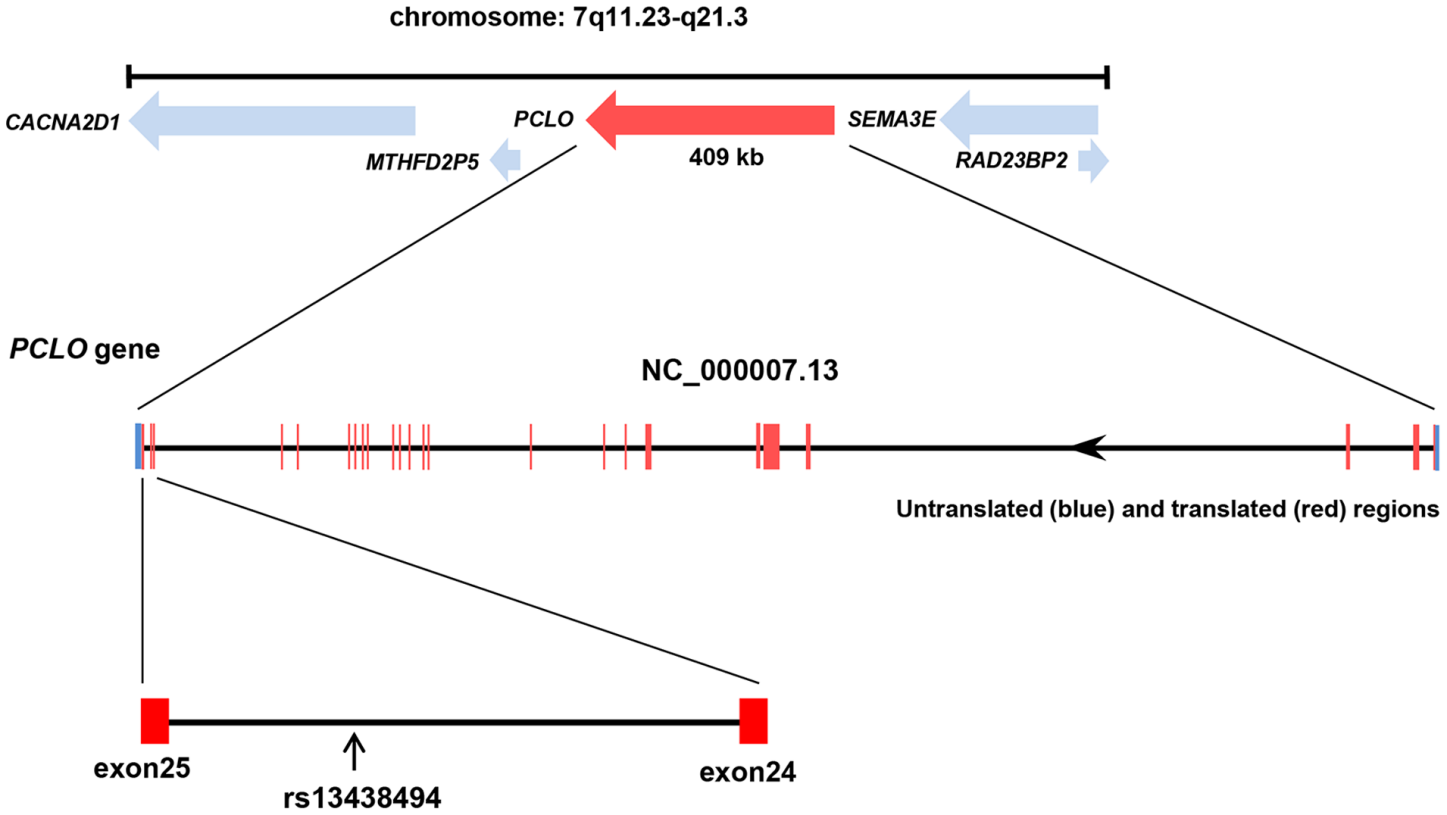
Physical map of PCLO gene locus and SNP rs13438494 location in *PCLO.* * PCLO* is located on chromosome 7 and transcribed in opposite direction. This gene spans 409 kb and comprises 25 exons. The position of rs13438494 in intron 24 of *PCLO* is indicated.

Human *PCLO* exon 24, intron 24, and exon 25 were amplified by PCR from human genomic DNA (Zyagen, USA). Primers were used to generate a fragment containing 146 bp of exon 24, 141 bp of exon 25, and 1923 bp of intron 24 (Table 1). We tailed the forward primer with XhoI (Takara, Japan) and the reverse primer with BamHI (Takara, Japan) to facilitate the cloning. After the confirmation of successful amplification through the detection of the expected 2210-bp band on an agarose gel, the products were digested with XhoI and BamHI (Takara, Japan) restriction enzymes and directly ligated into the XhoI/BamHI restriction points of the GFP expression vector pAcGFP-C2 vector (Clontech-BD Biosciences, USA). Ligation into pAcGFP vector was performed at room temperature for 1 h using T4 DNA ligase (Takara, Japan). *E. coli* JM109 competent cells (Toyobo, Japan) were transformed with the plasmid constructs and plated overnight. The sequences of the resulting clones were checked. Minigene constructs were isolated using a midiprep kit (Qiagen, Germany). The resulting pAcGFP-*PCLO* minigene constructs are shown in Figure 1. Single nucleotide substitution was introduced by oligonucleotide site-directed mutagenesis using TaKaRa Primestar polymerase (Takara, Japan). The mutagenic primer pairs were used to generate the nucleotide substitutions as indicated in bold (Table 1). The mutated construct was sequenced to confirm that only the desired change was introduced, and the construct was then isolated with a midiprep kit (Qiagen, Germany). The minigene constructs containing either A or C alleles were transfected into SH-SY5Y cells.

**Table 1:** Primers used for cloning of the *PCLO* minigene and site-directed mutagenesis.

	Primer sequence
**Cloning of ** ***PCLO*** ** minigene**	Forward: 5′ CCGCTCGAGCATTTATATGTGAAAATATATGTGATG 3′
(Exon 24 to 25)	Reverse: 5′ CGGGATCCTCAATGCGTTTGAGTAGGACTGA 3′
**A to C substitution**	Forward: 5′ GGAAGTACAAATTTTGAAGT**C**AGAAGCATAAAAGTTTTCGCC 3′
	Reverse: 5′ GGCGAAAACTTTTATGCTTCT**G**ACTTCAAAATTTGTACTTCC 3′

Restriction site targets introduced to allow sequential cloning of the PCR-amplified fragments are underlined. The nucleotide replaced by site-directed mutagenesis is indicated in bold.

### Cell Culture and Transfection

SH-SY5Y cells were obtained from the American Tissue Culture Collection (ATCC) and used within 10 passages of the original vial. SH-SY5Y cells were grown in DMEM/Ham’s F12 medium **(**Wako Pure Chemicals, Japan) supplemented with 10% fetal bovine serum (FBS) and 1% penicillin/streptomycin. Cell cultures were all maintained at 37°C in a humidified atmosphere containing 5% CO_2_.

The *PCLO* minigene constructs were transiently transfected into SH-SY5Y cells using Lipofectamine 2000 (Invitrogen, USA) according to the manufacturer’s recommendations. In brief, cells were grown to 80% confluency in 12-well plates for 24 h in complete growth medium without antibiotics and exposed to a mixture of 2 µl/well of lipofectamine and 0.8 µg/well of plasmid DNA. Cells transfected with the empty AcGFP vector were used as controls. At 48 h after transfection, the cells were harvested, and total RNA was extracted for RT-PCR. Transfection efficiency was monitored by checking and counting GFP-fluorescent cells under an optic fluorescence microscope (Carl Zeiss, Germany).

### RT-PCR of the *PCLO* Minigene for *in vitro* Splicing Assays

Total RNA from transfected SH-SY5Y cells was extracted using TRIsure (Bioline, UK) following the manufacturer’s instructions. A 500 ng aliquot of total RNA was used to generate first strand cDNA using PrimeScript™ RT reagent Kit (Takara, Japan). To evaluate the pattern of transcripts produced from the transfected constructs, the following vector-specific primers were used for RT-PCR amplification: a pAcGFP Fw primer (5′-CCGACCACTACCAGCAGAAT-3′) and a SV40pA Rv primer (5′-GAAATTTGTGATGCTATTGC-3′). *GAPDH* was used as an internal control: a forward primer (5′-CCACCCAGAAGACTGTGGAT-3′) and a reverse primer (5′-CCCTGTTGCTGTAGCCGTAT-3′). Amplified products were separated by agarose gel electrophoresis, and each band signal was quantified by ImageJ software (NIH, USA). All transcripts were further analyzed by excising the bands from the gel by means of a Gel Extraction Kit (Qiagen, Germany) and subsequent direct sequencing.

### *In silico* Splicing Analysis

The following web-based tools were used for splice-site analysis: ASSP (http://wangcomputing. com/assp/), NetGene2 (http://www.cbs.dtu.dk/services/NetGene2), NNSplice (http://www.fruitfly. org/seq_tools/splice.html), Human Splicing Finder (HSF) (v.2.4) (http://www.umd.be/HSF/), MaxEntScan (http://genes.mit.edu/burgelab/maxent/Xmaxentscan_scoreseq.html), and SpliceView (http://zeus2.itb. cnr.it/_webgene/wwwspliceview_ex.html). Human default parameter settings were used in all analyses. These programs were used to scan the entire sequence of the human *PCLO* minigene, and splice sites and their scores were compared.

In addition, the HSF matrices were used to analyze the effect of rs13438494 on putative splicing regulatory sequences. HSF integrated all available matrices to identify exonic and intronic motifs: exonic splicing enhancer (ESE) motifs from HSF (ESE-HSFs) for serine/arginine-rich (SR) proteins (SRp40, SC35, SF2/ASF, SF2/ASF IgM/BRCA1, and SRp55), RESCUE ESE hexamers (RESCUE-ESE), putative 8-mer ESEs (PESEs) and putative 8-mer exonic splicing silencers (PESSs), exon identity and intron-identity elements (EIEs and IIEs), heterogeneous nuclear ribonucleoprotein (hnRNP)–binding motifs, and Fas exonic splicing silencers (ESS).

## Results

**Figure 2:**
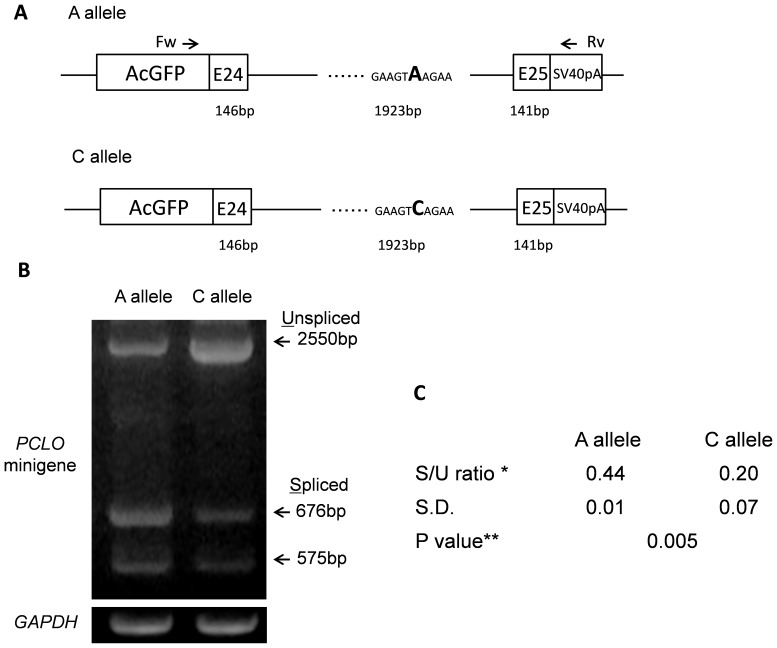
Construction and expression analysis of the *PCLO* minigene. (A) Representation of the *PCLO* minigene fused to the GFP coding sequence. The position of the mutation is indicated in bold. The positions of the primers used for RT-PCR are indicated by small arrows. (B) RT-PCR of cells transfected with the *PCLO* minigene constructs. The upper band denotes the unspliced transcript. The central band corresponds to a 676-bp spliced product. The lower band denotes a 575-bp alternative product. *GAPDH* was used as the control for normalization. (C) Splicing efficiency of the spliced and unspliced transcripts from three independent experiments. *Transcript splicing efficiency is the mean ratio of fluorescent intensity of correctly spliced transcripts: spliced plus unspliced transcripts for a given expression construct. Standard deviations from three independent experiments are shown. **Two-tailed P value calculated by Student’s t-test.

The SNP rs13438494 (*c.15289-683A>C*) in *PCLO* is an A>C transition located deep in intron 24. The human Piccolo gene compasses 25 exons spanning 409 kb of genomic DNA, and it maps to 7q11.23-q21.3, a region of chromosome 7 (Fig. 1). We generated minigene constructs containing exon 24, intron 24, and exon 25 and the cytomegalovirus (CMV)-driven GFP gene fused in-frame upstream of exon 24 (Fig. 2A).

To investigate the role of this SNP in regulating splicing, we performed *in vitro* experiments that tested the splicing patterns associated with the A and C alleles (Fig. 2B). To avoid amplifying the endogenous *PCLO* gene, we used the vector-specific primers AcGFP Fw and SV40pA Rv for RT-PCR and exclusively amplified the transcripts produced by the minigene. *GAPDH* was used as a control for RNA quantity and quality. Each band was quantified by ImageJ (NIH, USA). RT-PCR of the minigene construct containing the C allele yielded the same three bands as that containing the A allele, but in different proportions, with both unspliced and spliced transcripts being produced (Fig. 2C). After gel extraction, each PCR product was directly sequenced. The upper band of approximately 2.6 kb corresponded to unspliced transcripts including exon 24, exon 255, and the intronic flanking sequences. The 676-bp fragment included exons 24 and 25 as expected, and the third fragment of 575 bp matched with the exons 24 and 25 but lacked the last 101 nucleotides of exon 24. The transcript lacking the last 101 nucleotides of exon 24 was expressed in both minigene constructs. To investigate the skipping of the final 101 bp of exon 24, the entire sequence of the *PCLO* minigene was scanned by different programs for splice site prediction analysis (for details see Materials and Methods). Although each program calculates the score according to a different algorithm, all analyses gave similar results (data not shown). The analysis of splice site prediction revealed that the site skipped the last 101 bp of exon 24 had a very high score as a cryptic splicing donor, and in a minigene assay, activation of the site as a splicing donor site resulted in skipping of the last 101 bp of exon 24. Our result of an increase in expression of the retained intron and a decrease in constitutive exon expression linked with the C allele of rs13438494 suggested that this allele reduces the splicing efficiency of the *PCLO* minigene.

We next focused our attention on the possible mechanism underlying the observed aberrant splicing. We attempted to further characterize rs13438494 in *PCLO* through *in silico* analysis (Table 2). Bioinformatics analysis of potential splicing aberrations was performed using HSF (v.2.4). The majority of the algorithms used for the prediction of enhancer/silencer motifs by HSF (v.2.4) web tool indicated that rs13438494 induces the abrogation or creation of such binding sites. Thus, these data suggested that the reduced splicing efficiency may be caused by the disruption/creation of an enhancer/silencer motif, which is created by the SNP rs13438494.

**Table 2:** *In silico* analysis of the SNP rs13438494 located in intron 24 of *PCLO.*

		Motif	Motif		
	Method	A allele	C allele	Threshold	Result
		(Value 0–100)	(Value 0–100)		
ESE Finder		–	T**C**AGAAG(86.29)	78.08	New SRp40 binding site
Rescue ESE		GAAGT**A**	–	–	Enhancer motif site broken
		–	T**C**AGAA		New enhancer motif site
PESE Octamers		GAAGT**A**AG(24.84)	–		Enhancer motif site broken
(Zhang & Chasin)		**A**AGAAGCA(36.88)	–	–	Enhancer motif site broken
EIEs (Zhang et al)		–	GT**C**AGA	–	New enhancer motif site
ESE from HSF		GAAGT**A**(68.52)	GAAGT**C**(80.60)	59.245	9GB value increase
		T**A**AGAA(66.18)	–	59.245	9GB site broken
		**A**AGAA(100)	–	75.964	Tra2-β site broken
Silencer motifs		T**A**AGAAGC(65.82)	T**C**AGAAGC(63.60)	60	Motif 1 value decrease
(Sironi et al)					
hnRNP motifs		T**A**AGAA	–	65.476	hnRNP A1 binding site broken(74.53)
Other splicing motifs		GAAGT**A**		–	Motif sites borken
(Goren et al)		**A**AGAAG		–	Motif sites borken
		–	T**C**AGAA		New motif site

The allele of rs13438494 is indicated in bold.

These algorithms are included in the analysis of Human Splice Finder (v.2.4).

## Discussion

SNP rs13438494 (*c.15289-683A>C*), which is reported to be significantly associated with bipolar disorder in a recent meta-analysis of GWAS [13], [14], is located deep in intron 24 of *PCLO*. Despite the fact that introns comprise >90% of the sequence of a gene, most reported mutations are located in exonic sequences. However, there are an increasing number of new pathogenic variants located in introns. Recently, many disease-related mutations have been reported to be responsible for aberrant splice processes [15], [16]. Most of the mutations affecting splicing disrupt the highly conserved donor and acceptor sites (GT/AG) at exon-intron junctions, the polypyrimidine tract, and the branch-point sequence with different consequences such as exon skipping and the activation of cryptic splice sites. However, attention is now being drawn to mutations deep in intronic sequences that affect the less well-conserved auxiliary splicing sequences–that is, exonic and intronic splicing enhancers or silencers–that help in the recognition and binding of specific splicing regulatory proteins such as SR proteins and RNP [17]. We hypothesized that this intronic SNP may modulate the splicing efficiency of the *PCLO* minigene.

To investigate whether a deep intronic mutation, rs13438494, in *PCLO* affects splicing, we constructed minigenes with A and C alleles containing exon 24, intron 24, and exon 25 and transfected them into human SH-SY5Y cells. The human neuroblastoma cell line SH-SY5Y is a subclone of the parent cell line SK-N-SH, which was originally established from a bone marrow biopsy of a neuroblastoma patient. SH-SY5Y cell line has been commonly used to investigate the molecular and cellular functions of identified susceptibility genes for psychiatric disorders because they endogenously express neural proteins [18]. Semi-quantitative RT-PCR analysis using vector-specific primers for minigene constructs revealed the presence of three transcripts, an unspliced transcript including intron 24, a spliced transcript containing exon 24 and exon 25, and a spliced transcript lacking the last 101 nucleotides of exon 24, indicating an in vitro equilibrium among the splicing products. The ratio of unspliced to spliced transcripts was higher in samples with the C allele than in those with the A allele. Our *in vitro* experiments indicate that the relative decreased abundance of the spliced form (the level of *PCLO* minigene activity) is affected by the intronic SNP rs13438494.

The spliced *PCLO* minigene isoform lacking the last 101 nucleotides of exon 24 was expressed in both minigenes constructs, although it was expressed at a very low level. Splice site prediction analysis using different web tools demonstrated that the site that resulted in the skipping of 101 bp had a very high score as a cryptic splicing donor. The 101 bp-skipped band has not been previously reported as a splicing variant of *PCLO* in previous studies [19]. Thus, in a minigene assay, activation of the site as a splicing donor resulted in skipping of the last 101 bp of exon 24.

An unspliced product was also observed in both minigene constructs. However, potential plasmid DNA contamination in the cDNA templates as the source of the unspliced bands was excluded because no amplification of genomic DNA was observed from the templates for RT-PCR of *GAPDH* with primers designed to span the introns of the genomic sequence. This transcript has not been reported previously as a splicing variant of *PCLO* in previous studies [19]. The transcript with an unspliced intron was produced due to the premature termination of translation by introducing a stop codon in the *PCLO* minigene [20]. The relative amount of the unspliced form was significantly greater in C allele. An increase in the number of such immature transcripts may lead to reduced activity of the *PCLO* minigene. Therefore, we speculate that the reduced splicing associated with the C allele could result in lower spliced transcript levels for the *PCLO* minigene.

This deep intronic mutation is not located in the coding sequence or near the constitutive splice sites, but it may influence the splicing process through the abrogation/creation of enhancer/silencer motifs. Therefore, we next performed an *in silico* analysis to investigate whether aberrant splicing due to rs13438494 is caused by creating or disrupting the splicing enhancer or silencer [21]. The search for potential splicing regulatory elements using various algorisms indicated that rs13438494 induces the abrogation or creation of such binding sites. Interestingly, the C allele creates a new high score of SRp40 motif (86.29) bound to splicing enhancer and abolishes an hnRNP motif bound to splicing silencer.

Altogether, these data suggested that the deep intronic SNP rs13438494 (*c.15289-683A>C*) reduced the splicing efficiency of the *PCLO* minigene by creating a splicing enhancer and/or abolishing a splicing silencer, which potentially could participate in the binding of splicing regulatory proteins. The alternation of splicing efficiency by SNP rs13438494 may ultimately influence bipolar disorder. There are recent advances in strategies in modulating splicing therapeutically in clinical and preclinical contexts [22]. The molecular basis for individual variation in splicing efficiency is largely unknown, but identification of the responsible factors could facilitate the development of therapies. We have been particularly interested in *PCLO* as a factor for understanding mental disorders. *PCLO* encodes a protein that is localized to the presynaptic active zone and plays an important role in monoaminergic neurotransmission in the brain. The physiological impact of this SNP on monoamine transmission requires further study.
